# Electrical equivalent circuit for analyzing the effect of signal shape on power distribution in cochlear implant electrodes and surrounding tissue

**DOI:** 10.1038/s41598-025-04840-5

**Published:** 2025-06-20

**Authors:** Merle Sehlmeyer, Mariia Makarenko, Nele Schoerner, Mit B. Bhavsar, Tatiana Blank, Hans Jürgen Maier, Andrej Kral, Hannes Maier, Stefan Zimmermann

**Affiliations:** 1 Institute of Electrical Engineering and Measurement Technology, Leibniz University Hannover, Appelstr. 9A, 30167 Hannover, Germany; 2 Department of Otolaryngology/NIFE and Cluster of Excellence “Hearing4all”, Hannover Medical School, Stadtfelddamm 34, 30625 Hannover, Germany; 3 Institut für Werkstoffkunde (Materials Science), Leibniz University Hannover, An der Universität 2, 30823 Garbsen, Germany; 4 Department of Experimental Otology/NIFE, Hannover Medical School, Stadtfelddamm 34, 30625 Hannover, Germany

**Keywords:** Cochlear implant, Impedance spectroscopy, Electrical equivalent circuit, Electrode–electrolyte interface, Numerical signal analysis, Biomedical engineering, Electrical and electronic engineering

## Abstract

Cochlear implants are a well-established solution for restoring hearing in severe impairment and profound deafness. However, cochlear implants still have limitations, such as speech recognition in noisy environments caused by intra-cochlear current spread across different auditory spiral ganglion neurons as a consequence of, e.g., the large distance of the stimulation electrodes to the target cells in a highly conductive environment. Stimulation in cochlear implants is typically done with charge balanced biphasic rectangular current pulses in a monopolar arrangement. However, several studies have shown that a rectangular stimulation pulse is not optimal for stimulating spiral ganglion neurons. For example, stimulation with a ramped pulse, such as a sawtooth pulse, has been shown to be more energy-efficient and achieves a similar threshold profile in spiral ganglion neurons. In this study, a new but simple equivalent electrical circuit model is introduced that describes the complex impedance between two stimulation electrodes of a cochlear implant with high accuracy (mean relative error ≤ 8%). Based on this bipolar model, a monopolar equivalent electrical circuit model is developed to describe the stimulation between one stimulation electrode and a counter electrode located outside the cochlea. These two models now allow for analyzing the effect of stimulation pulse shape on power distribution in cochlear implant electrodes and surrounding tissue providing a tool for investigating stimulation efficiency with respect to energy losses in the cochlear implant electrode.

**Supplementary Information:**

The online version contains supplementary material available at 10.1038/s41598-025-04840-5.

## Introduction

Cochlear implants (CIs) are an established solution for restoring hearing in severe hearing loss to profound deafness. However, CIs still have limitations, such as speech recognition in noisy environments caused by, e.g., intra-cochlear current spread across different auditory spiral ganglion neurons (SGNs)^1–3^. Stimulation in CIs is typically performed with biphasic, rectangular current pulses in a monopolar arrangement^4^ between an intra-cochlear stimulation electrode (SE) located inside the cochlear duct and an extra-cochlear counter electrode located outside the cochlea, e.g., under the scalp to avoid unwanted electrochemical reactions^5^. However, various studies have already shown that a rectangular stimulation pulse is not optimal for stimulating the SGNs^6–8^. When stimulating with a rectangular pulse, the neighboring SGNs are also stimulated with a rectangular pulse even though the current amplitude decreases with increasing distance from the stimulation center, as shown by Ballestro et al. in vitro^6,7^.

This current spread is a known issue of CIs caused by the inherent design of the small stimulation electrodes with large distance of the SEs to the SGNs in highly conductive perilymph, leading to an unwanted spatially extended activation of SGNs, overlapping stimulation of neighboring channels and reduced frequency discrimination^6,9–11^. When stimulation is performed with a ramped pulse shape, such as a sawtooth pulse, both the current amplitude and the edge steepness of the pulse decrease with increasing distance from the stimulation center^6^. This results in fewer neighboring SGNs being unintentionally stimulated (see Figure 3 in^6^). Furthermore, Navntoft et al. have shown in vivo that ramped pulses with less edge steepness are significantly more energy-efficient than a rectangular stimulation pulse with steep edges and achieve a response of SGNs with similar threshold profile in mice and human CI users^7,12^. Also, Yip et al. have investigated arbitrary pulse shapes in vivo in human cadaveric temporal bones and have found 15–35% power saving compared to the conventional rectangle pulse shape^13^. The aim of this study is to build on these findings and analyze the effect of stimulation pulse shape on power distribution in CI electrodes and surrounding tissue by employing an accurate equivalent electrical circuit (EEC) model of the CI electrodes. While most existing EEC models, such as that of Vanpoucke et al., model the bilayer with linear, frequency-independent electrical elements^14–17^, this study uses a non-linear, frequency-dependent polarization capacitance and a non-linear, frequency-dependent polarization resistance to model the bilayer between the SEs and the electrolyte more realistically. Nevertheless, there are already EEC models, such as that of Jiang et al., that use constant phase elements (CPE) to model the frequency-dependent behavior of the bilayer^11^. However, our previous study has shown that the Schwan-Faraday model^18^ used for the bilayer in this study can achieve even better representation of the results from impedance spectroscopy of CI electrodes in bipolar configuration than a CPE model^19^. In addition, the EEC presented here also integrates the impedance characteristics of the connections within the CI electrode carrier, which affect the impedance measurement and thus need to be considered to accurately represent the impedance behavior of the entire CI electrode.

In addition to current spread reducing frequency selectivity, other issues can limit the stimulation efficiency of CIs. Different studies have shown, how impedance measurements can help to detect misplacement of the CI electrode in the cochlea during insertion^20^, electrode migration^21,22^, bleeding caused by injury^23^ or fibrocyte growth on the SEs^24^, all affecting stimulation efficiency. Also, in commercially available CIs, impedance measurements already serve multiple diagnostic purposes, e.g., the detection of tip-fold-over^25^ or determination of full insertion^26^. In clinical use, individual stimulation electrode impedances are typically measured monopolar between the intra-cochlear SEs and the extra-cochlear counter electrode either at the beginning or at the end of a rectangular stimulation pulse, depending on the manufacturer^4,27^. Compared to a monopolar measurement between one SE in the cochlea and a counter electrode located outside the cochlea, bipolar measurements between two SEs offer the advantage of short pathways between the two active SEs, and thus provides information about the local environment of these SEs and the surrounding tissue^20^. Additionally, there are other impedance measurement configurations for cochlear implants worth mentioning. For example, three-pole impedance measurements use a current stimulation between one intra-cochlear SE and an extra-cochlear reference, while measuring the voltage between two other intra-cochlear SEs, including the unknown current pathway to the reference^20^. In four-pole impedance measurement configuration, stimulation current is induced between two intra-cochlear SEs that are further apart, while measuring the voltage across two of the other intra-cochlear SEs in between^23^. However, in four-pole configuration precise impedance measurement requires knowledge of the current density between the voltage measuring SEs and both configurations usually do not consider the connection lines of the CI. To exploit the full potential of impedance measurements, impedance spectroscopy over a wide frequency range is suggested^11^. This allows for a more accurate electrical characterization of the CI electrode and its electrochemical behavior in interaction with the surrounding tissue and perilymph.

In the present study, a simple non-linear and more detailed EEC was developed that models the bipolar impedance between all pairs of SEs with a mean relative error below 8%. For an experimentally validation of this EEC, bipolar impedance measurements were conducted using sinusoidal voltage excitation in the frequency range from 20 Hz to 20 MHz between the most apical SE and all other SEs characterizing an HiFocus SlimJ cochlear implant array from Advanced Bionics regarding its frequency-dependent electrical and electrochemical behavior and interaction with the surrounding tissue. The new EEC is based on a previously published EEC, which describes the impedance of neighboring SEs^19^, whereas the new extended bipolar EEC is able to describe the impedance for any possible SE combination due to an extension to include the electrical pathway through the surrounding material and the wire inductivity. Based on this new validated bipolar EEC, a monopolar EEC is developed to model the stimulation between one SE and a counter electrode located outside the cochlea. Both EEC models are intended to characterize the electrical properties of CI electrodes before implantation in order to compensate for the electrode properties in later measurements, for example to draw conclusions about the position of the CI electrode inside the cochlea or to detect cell growth on the SEs. Furthermore, these in silico models allow for analyzing the effect of different stimulation pulses on the power distribution over different components of the CI electrode and thus energy efficiency of CI and the power input into the surrounding tissue.

## Materials and methods

To develop and validate the EEC, in this study, bipolar impedance measurements were carried out between two SEs of a HiFocus SlimJ CI electrode provided by Advanced Bionics LLC (AB, Valencia, CA, USA). The CI electrode was connected to a small custom-made printed circuit board (PCB) for reproducible connection to an impedance analyzer. The CI electrode featured an SE array with 16 SEs, each SE connected with the PCB via the thin platinum connecting wires curled in spiral shape inside the silicone. Each platinum wire has an individual ohmic resistance proportional to its length^19^. Particularly relevant for bipolar impedance spectroscopy is the formation of a capacitance between the wires of the active SEs, which leads to crosstalk^19^. The curling of the wires leads to an additional inductivity per wire, which was determined experimentally with an LCR meter (R&S®HM8118 LCR Bridge/Meter, R&S HM8118, Rohde & Schwarz GmbH & Co KG, Germany) and can be found in Fig. S1 in the supplementary information. Consequently, the wire inductances, capacitances and resistances are included in the EECs where applicable.

### Impedance measurement

Before each impedance measurement, the CI electrode was cleaned in a solution of the enzyme-active detergent Tergazyme® (cat. No. 1304-1, Lot MKCM5800, Alconox Critical Cleaning Experts, New York, USA) for 10 min to remove any contamination and to ensure reproducible measurements. Residual solvent on the CI electrode was removed afterwards by placing the CI electrode in distilled water for 10 min. The cleaned CI electrode was connected to an E4990A impedance analyzer (Keysight Technologies Inc., Santa Rosa, CA, USA) using test fixture 16047A (Keysight Technologies Inc., Santa Rosa, CA, USA). The impedance analyzer was calibrated performing a short and open measurement as well as a load measurement with a 50 Ω resistor at the end of the test fixture according to the impedance analyzer’s manual^28^. After calibration, the CI electrode was placed in a 3D printed epoxy cylinder (Bio-Med Clear, Liqcreate, Utrecht, Netherlands) with an outer diameter of 25 mm and a height of 35 mm. In the center of the circular end of the cylinder was a blind hole with a diameter of 1.2 mm and a depth of 25 mm filled with 0.9% saline of 16.06 mS/cm conductivity (B. Braun SE, Melsungen, Germany) giving a simple, straight cochlea phantom filled with perilymph. After placing the CI electrode in the cochlea phantom, impedance spectroscopy was performed between 20 Hz and 20 MHz with logarithmic sweeping (1601 points) at a voltage of 100 mV_rms_.

### Electrical equivalent circuit for bipolar arrangement (SE vs. SE)

**Fig. 1:**
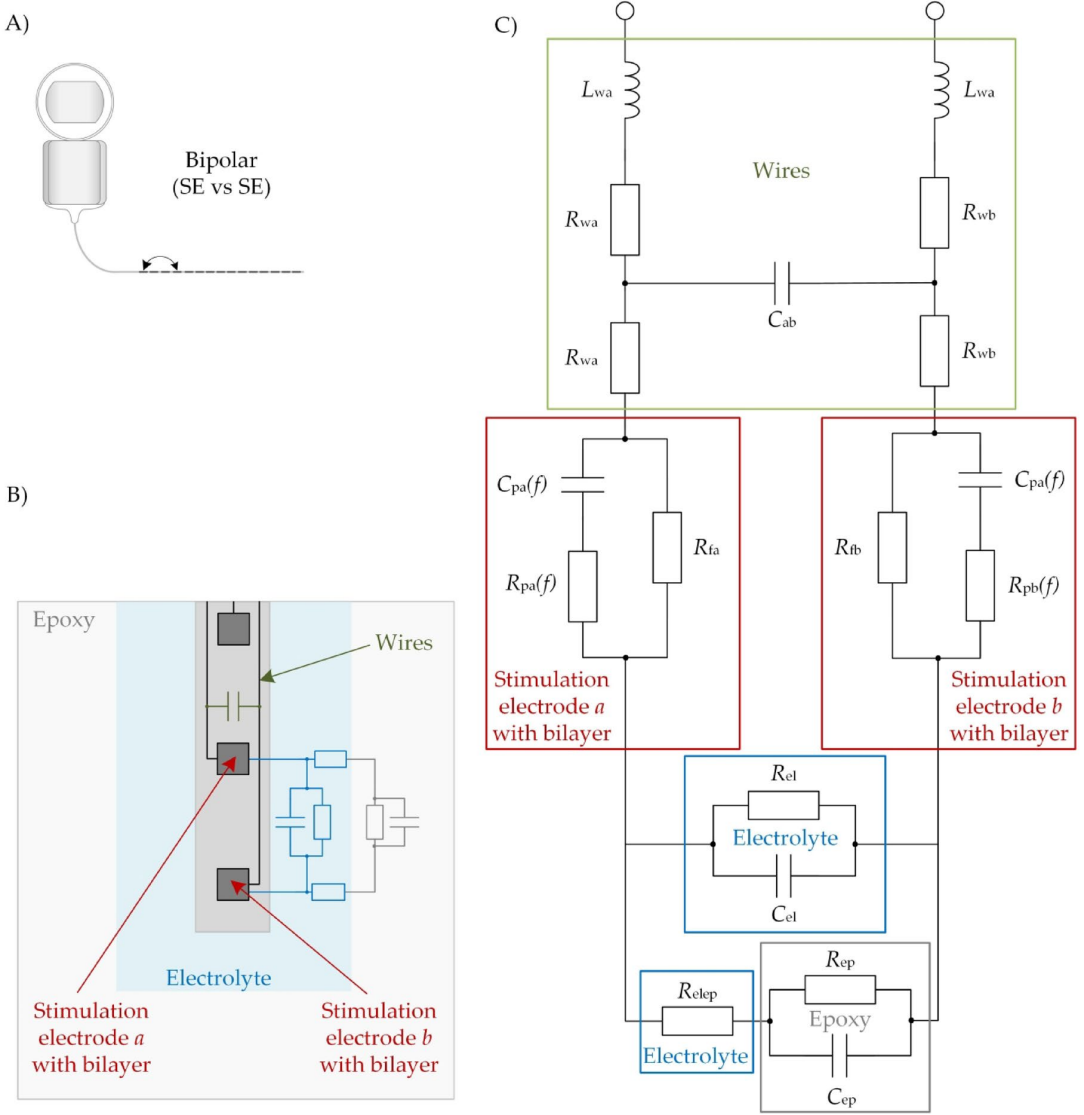
(**A**) Schematic depiction of a bipolar stimulation (adapted from Sijgers et al.^20^). (**B**) Schematic depiction of a CI electrode placed in a saline filled epoxy cylinder. (**C**) Proposed bipolar EEC of two SEs (*a* and *b*) of a CI. The equivalent electrical circuit consists of the connecting wires inside the silicone carrier (green, wire inductances $L_{{{\text{wa}}}}$, $L_{{{\text{wb}}}}$, wire resistances $R_{{{\text{wa}}}}$, $R_{{{\text{wb}}}}$ and capacitance between wires $C_{{{\text{ab}}}}$), the SE-electrolyte interfaces (red, frequency-dependent polarization capacitances $C_{{{\text{pa}}}}$, $C_{{{\text{pb}}}}$, polarization resistances $R_{{{\text{pa}}}}$, $R_{{{\text{pb}}}}$ and faradaic resistances $R_{{{\text{fa}}}}$, $R_{{{\text{fb}}}}$), the electrolyte between the SEs (blue, resistance $R_{{{\text{el}}}}$ parallel to capacitance $C_{{{\text{el}}}}$ and resistance $R_{{{\text{elep}}}}$) and the surrounding epoxy cylinder (grey, resistance $R_{{{\text{ep}}}}$ parallel to capacitance $C_{{{\text{ep}}}}$).

Electrically a CI electrode consists of four different components as shown in the proposed bipolar EEC in Fig. 1—the wires (green), the interface between each SE and the electrolyte (red), the electrolyte between two SEs (blue) and the material surrounding the electrolyte and CI electrode (grey). Apart from the electrode array with the exposed SEs, the wires connecting the SEs are curled in spiral shape embedded in a silicone carrier, which leads to the inductances $L_{{{\text{wa}}}}$, $L_{{{\text{wb}}}}$. Additionally, each wire has a resistance ${R}_{\text{wa}}$, $R_{{{\text{wb}}}}$ and a capacitance $C_{{{\text{ab}}}}$ is formed between the wires. The SE-electrolyte interfaces can be described by the non-linear, frequency-dependent polarization capacitances $C_{{{\text{pa}}}}$ and $C_{{{\text{pb}}}}$, the non-linear, frequency-dependent resistances $R_{{{\text{pa}}}}$ and $R_{{{\text{pb}}}}$ in parallel to the faradaic resistances $R_{{{\text{fa}}}}$ and $R_{{{\text{fb}}}}$, already described by Schwan et al.^18^. Two active SEs are connected by the electrolyte resistively via $R_{{{\text{el}}}}$ and capacitively via $C_{{{\text{el}}}}$. Also, compared to our previous publication^19^ there is an additional connection between the SEs through the surrounding material and the inductances $L_{{{\text{wa}}}}$, $L_{{{\text{wb}}}}$. In the case of epoxy, the resistive coupling $R_{{{\text{ep}}}}$ is assumed as infinite in this study, while the capacitive coupling $C_{{{\text{ep}}}}$ shows a relevant effect in the measurements. The electrical pathway from the SEs through the electrolyte to the surrounding material is described with $R_{{{\text{elep}}}}$. Placing a CI electrode inside the cochlea, the resistive coupling through tissue and bone could be more relevant as these materials can be assumed to have a higher electrical conductivity than epoxy.

The wire parameters were measured for each SE individually. Initial parameters for the bilayer models, electrolyte and epoxy were estimated in MATLAB R2024a by analyzing the measurement data (see “Impedance measurement” section) as described in detail in our previous publication^19^ and subsequently optimized using the *fmincon* algorithm in MATLAB, minimizing the error function $\overline{e}_{{\underline {Z} }}$ of the complex impedance, see Eq. (3).

The bipolar EEC is evaluated using the mean absolute error (MAE) and mean relative errors (MRE) according to the Eqs. (1) to (3) as a measure of how well the modeled values for complex impedance $\underline {Z}_{{{\text{mod}}}}$, absolute value $\left| {\underline {Z} } \right|_{{{\text{mod}}}}$ and phase angle $\varphi_{{{\text{mod}}}}$ represent the measured complex impedance $\underline {Z}_{{{\text{meas}}}}$, absolute value $\left| {\underline {Z} } \right|_{{{\text{meas}}}}$ and phase angle $\varphi_{{{\text{meas}}}}$ averaged across all frequencies $f_{i}$ with $i = 1, \ldots N$^19^.

1 $$MAE\;of\;phase\;angle{:}\; \overline{e}_{{{\upvarphi }}} = \frac{1}{N}\mathop \sum \limits_{i = 1}^{N} \left| {\varphi_{{{\text{mod}}}} \left( {f_{i} } \right) - \varphi_{{{\text{meas}}}} \left( {f_{i} } \right)} \right|$$

2 $$MRE\;of\;absolute\;value{:} \;\overline{e}_{{\text{Z}}} = \frac{1}{N}\mathop \sum \limits_{i = 1}^{N} \frac{{\left| {\left| {\underline {Z}_{{{\text{mod}}}} \left( {f_{i} } \right)} \right| - \left| {\underline {Z}_{{{\text{meas}}}} \left( {f_{i} } \right)} \right|} \right|}}{{\left| {\underline {Z}_{{{\text{meas}}}} \left( {f_{i} } \right)} \right|}}$$

3 $$MRE\;of\;complexe\;impedance{:}\; \overline{e}_{{\underline {Z} }} = \frac{1}{N}\mathop \sum \limits_{i = 1}^{N} \frac{{\left| {\underline {Z}_{{{\text{mod}}}} \left( {f_{i} } \right) - \underline {Z}_{{{\text{meas}}}} \left( {f_{i} } \right)} \right|}}{{\left| {\underline {Z}_{{{\text{meas}}}} \left( {f_{i} } \right)} \right|}}$$

Additionally, the arithmetic means $\overline{{\overline{e}_{{{\upvarphi }}} }}$, $\overline{{\overline{e}_{{\text{Z}}} }}$ and $\overline{{\overline{e}_{{ \underline {Z} }} }}$ and standard deviations $\sigma_{{{\overline{\text{e}}}{\upvarphi }}}$, $\sigma_{{{\overline{\text{e}}\text{Z}}}}$ and $\sigma_{{{\overline{\text{e}}}{{\underline {Z} }} }}$ of all SEs measured against SE 1 were calculated from the averaged errors across all frequencies.

### Electrical equivalent circuit for monopolar arrangement (SE vs. counter electrode)

**Fig. 2:**
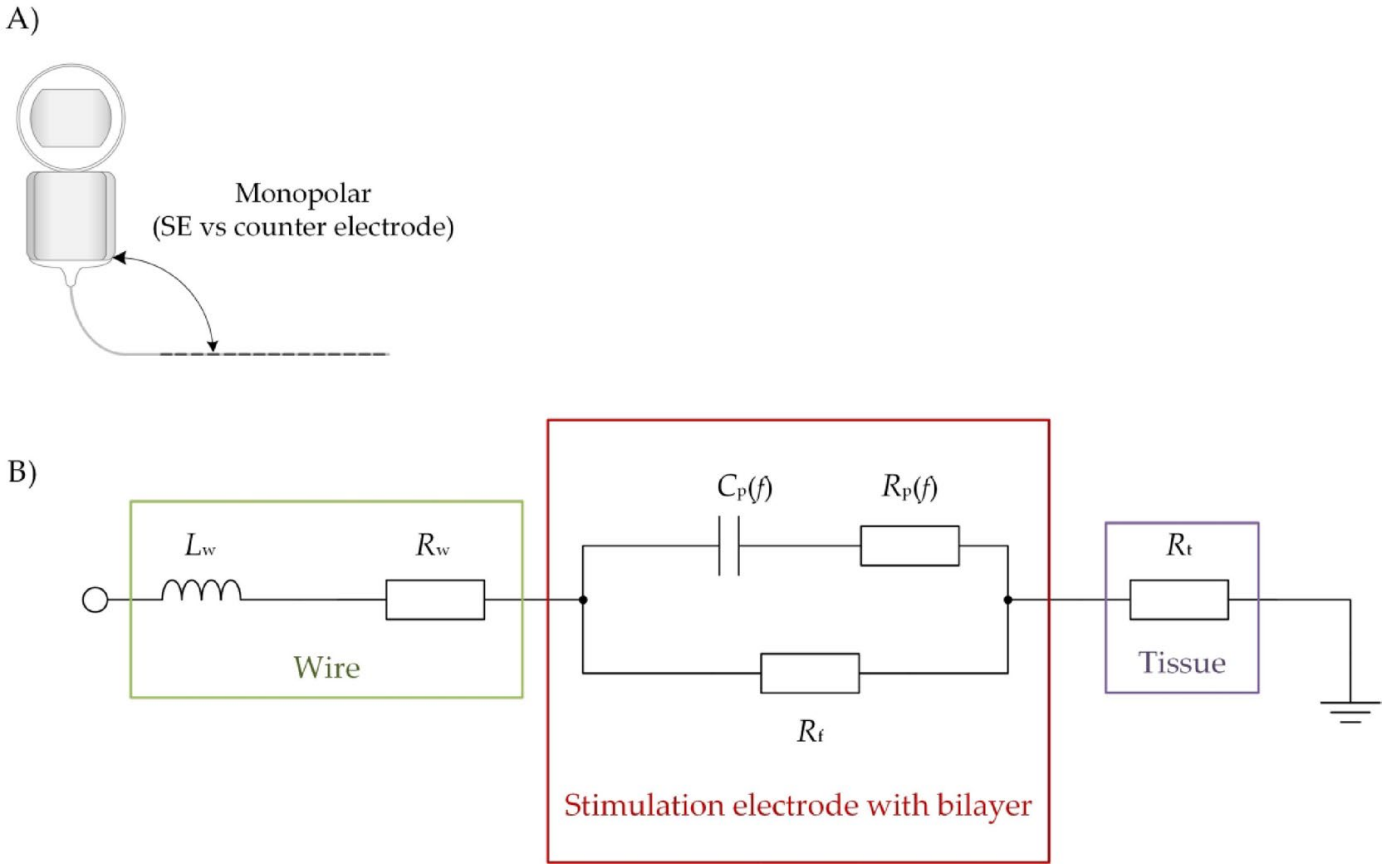
(**A**) Schematic depiction of a monopolar stimulation (adapted from Sijgers et al.^20^). (**B**) Proposed monopolar EEC for the monopolar stimulation with a SE against a counter electrode of a cochlear implant. The equivalent circuit consists of the connecting wires inside the silicone carrier (green, wire inductance $L_{{{\text{w}}}}$ and resistance $R_{{{\text{w}}}}$), the electrode–electrolyte interface (red, non-linear, frequency-dependent polarization capacitance $C_{{{\text{p}}}}$ and non-linear, frequency-dependent resistance $R_{{{\text{p}}}}$, faradaic resistance $R_{{{\text{f}}}}$ (as in the bipolar model in Fig. 1)) and the surrounding tissue (purple, resistance $R_{{{\text{t}}}}$).

**Fig. 3:**
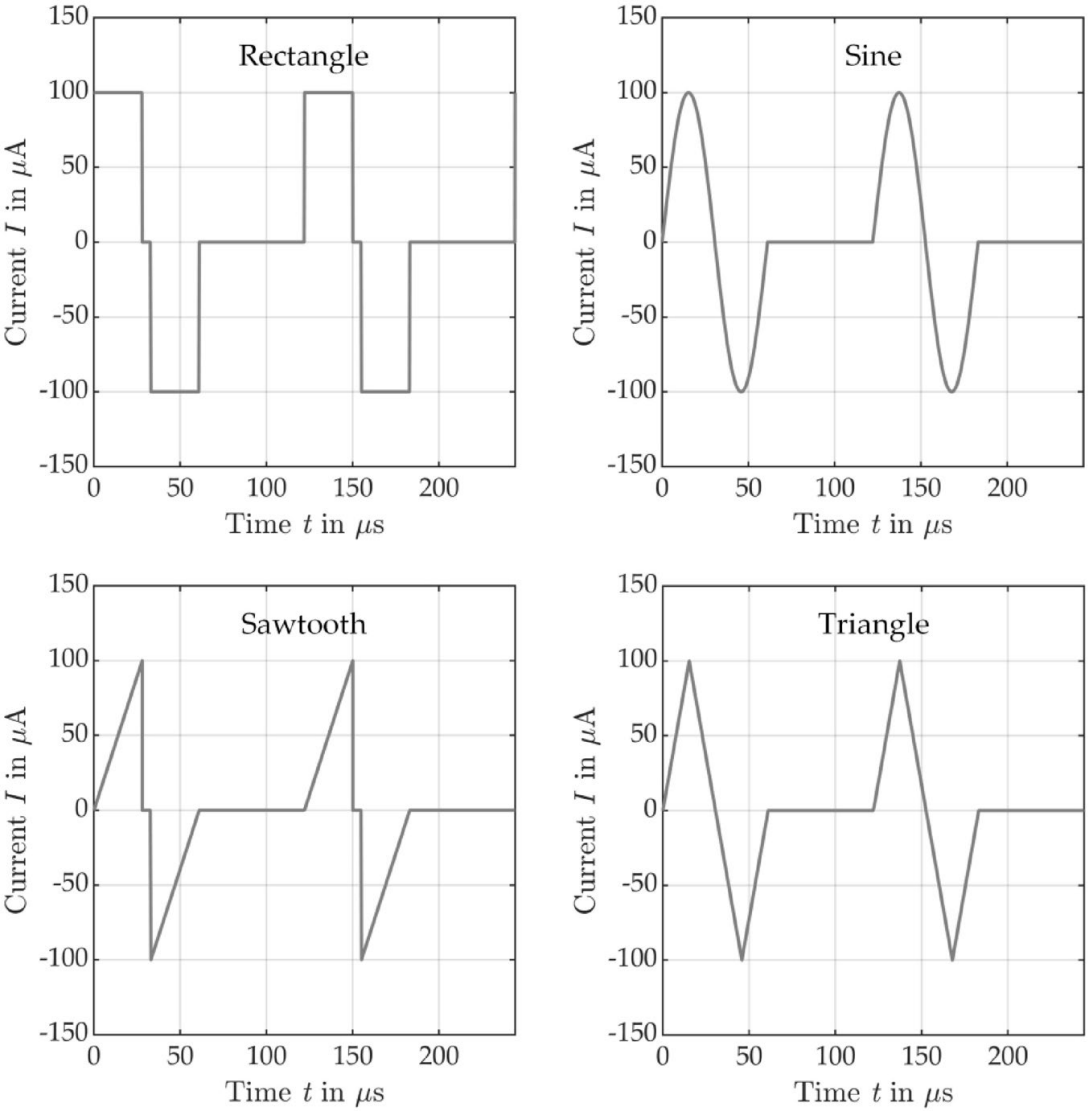
Biphasic stimulation current pulse shapes used in the analysis: rectangle, sine, sawtooth and triangle.

Based on the above bipolar EEC, an additional monopolar EEC was developed to describe the typical monopolar stimulation between one SE and a counter electrode (ground) located outside the cochlea. Therefore, the bipolar EEC was divided in the middle and extended by a return pathway to the counter electrode. The monopolar EEC is shown in Fig. 2 consisting of the wire of the SE (green), the SE with its bilayer (red) and the tissue (purple). The wire is characterized by an inductance $L_{{\text{w}}}$ in series with a resistance $R_{{\text{w}}}$. The SE with its bilayer is modeled the same way as in the bipolar EEC with a non-linear, frequency-dependent capacitance $C_{{\text{p}}}$ and a non-linear, frequency-dependent resistance $R_{{\text{p}}}$ parallel to a faradaic resistance $R_{{\text{f}}}$. This is different to existing EECs, which model the bilayer linear and frequency-independent^14–16^ as with linear and frequency-independent elements the EEC fails to represent the experimental results over a large frequency range. The electrical pathway between the SE and the counter electrode is characterized by the perilymph, bone and tissue around the CI electrode. In this model, this is simplified as a single tissue resistance $R_{{\text{t}}}$ neglecting a possible formation of a bilayer at the counter electrode, similar to the EEC by Aebischer et al.^14^. The possible capacitance between the SE and counter electrode is neglected in our monopolar EEC because of the large distance between these two electrodes of about 110 mm^29^. The parameters for wire and bilayer were assumed the same as in the bipolar EEC for one single SE. The tissue resistance to the counter electrode was estimated as 7.7 kΩ based on studies from Hu et al. who used impedance field telemetry in patients intraoperative and postoperative finding mean impedances 8 weeks after implantation of 7.7 kΩ for mid-array SEs (see Figure 3 in^30^). An evolution of impedance after implantation could also be observed by Wimmer et al. who found impedance values around 5 kΩ 12 months after implantation^31^. However, we assumed the tissue resistance with 7.7 kΩ. The clinical validation of the monopolar EEC would require clinical studies or investigations in cadaveric heads. Therefore, the EEC models in the present study were developed and validated using simple in vitro measurements in bipolar arrangement in combination with the mentioned tissue resistance of 7.7 kΩ.

### Numerical analysis of frequency-dependent CI behavior, stimulation signal shapes and power distribution across the different CI electrode components

Both EEC models (bipolar and monopolar) enable an in silico analysis of different stimulation pulse shapes regarding their voltage response and power distribution across the different components of the CI electrode and the surrounding tissue. To analyze the power distribution in dependence of the stimulating frequency, a sinusoidal stimulation voltage at different frequencies was applied to the EEC models giving the corresponding voltages across the individual components of the CI electrode—wires, bilayers and tissue or surrounding material. Multiplying these corresponding voltages and the current that has same value in all mentioned components due to the serial arrangement, results in the power received by the individual components and thus gives the power distribution. Therefore, the active SEs in the bipolar and the active SE in the monopolar EEC were excited with a continuous sinusoidal voltage of 100 mV_p_ at frequencies between 20 Hz and 20 MHz in MATLAB Simulink.

Subsequently, the power distribution of different time-dependent current pulse shapes with the same repetition rate was numerically analyzed regarding their power distribution across the different CI electrode and thus EEC components. First, both models were tested with a typical biphasic rectangular current pulse with a 28 µs long positive (anodic) phase, followed by a 5 µs pause and a subsequent negative (cathodic) phase of 28 µs. Each pulse was followed by a pause of 61 µs before the next pulse started. Since excitation with a rectangular pulse is not particularly energy-efficient due to its steep signal edges, the shape of the current pulse was adapted for further investigations. Stimulation with ramped pulses has already shown promising results^6,7,12^. Thus, a sawtooth with a rising edge was investigated, which has the same phase duration as the rectangular pulse. To avoid the steep signal edge at the end of the sawtooth pulse, also a triangle and sine pulse were examined. As these two pulse shapes do not have steep edges, there was no pause between the positive and negative pulse. The maximum amplitude of all current pulse shapes investigated was ± 100 µA. All current pulse shapes were biphasic and symmetrical (see Fig. 3, duration of one biphasic pulse: 61 µs) for charge balancing and compensation of faradaic reactions when later used in patients^5^.

## Results

In order to determine the optimum values of the EEC elements that best match the experimental data, impedance spectroscopic measurements between different SEs and the most apical SE in bipolar arrangement were used to align the characteristic of the EEC model with the experimental behavior by optimizing the initial EEC element values using the fitting routine *fmincon* in MATLAB. Afterwards, these EEC element values were used in the EEC models for a numerical, frequency- and time-dependent analysis of voltage response and power loss in dependence of signal shape.

### Impedance spectroscopy

**Fig. 4:**
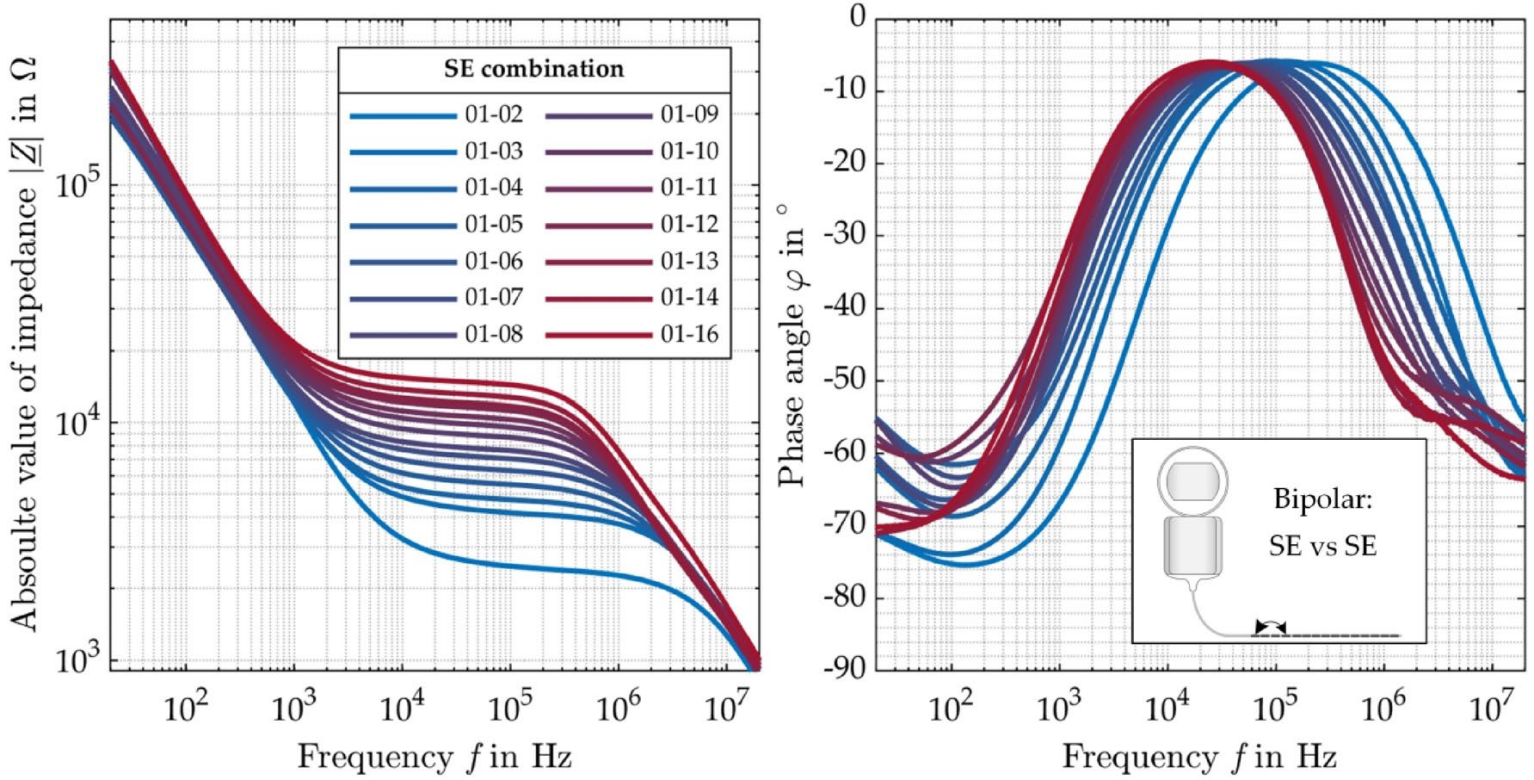
Absolute value $\left| {\underline {Z} } \right|$ (left) and phase angle $\varphi$ (right) of the complex impedance between the most apical SE (SE 1) and all other SEs (except SE 15, which was defect) of the AB HiFocus SlimJ electrode between 20 Hz and 20 MHz.

Figure 4 shows the bipolar impedance measurements of the most apical SE 1 against all other SEs. The recorded impedance spectrum can be divided roughly into three sections. In the low-frequency range (SE 1 vs. SE 2: 20 Hz to 10 kHz) the impedance shows capacitive behavior which is mainly dominated by the bilayers whereas in the medium frequency range (SE 1 vs. SE 2: 10 kHz to 3 MHz) the impedance behaves like a resistance that is mainly defined by the ohmic electrolyte. In the high frequency range (SE 1 vs. SE 2: 3–20 MHz), the impedance shows again capacitive behavior mainly caused by the cross capacitance between the wires connecting the active SEs. As expected, with increasing distance between the SEs, the absolute value of the complex impedance $\left| {\underline {Z} } \right|$ increases and the corner frequency shifts to lower frequencies (Fig. 4, left). This can be also observed in the phase angle plot where the maximum shifts to lower frequencies with increasing distance (Fig. 4, right). For frequencies above 1 MHz, a plateau like region can be observed in the phase angle plot, which is pronounced at large SE distance.

### Values of the elements of the bipolar EEC

Table 1 summarizes the measured elements representing the wires together with the subsequently optimized model values of the remaining elements of the bipolar EEC that best match the impedance experiment.

**Table 1:** Exemplary measured elements representing the wires together with values of the other elements of the bipolar EEC that best match the impedance experiment. The bilayer parameters for both SEs of a pair were assumed to be identical.

	SE 1 vs. SE 2	SE 1 vs. SE 6	SE 1 vs. SE 11	SE 1 vs. SE 16
Measured value				
${R_{{{\text{wa}}}}}$ (Ω)	61.55	61.55	61.55	61.55
${R_{{{\text{wb}}}}}$ (Ω)	62.65	60.00	56.85	53.75
${C_{{{\text{ab}}}}}$ (pF)	9.85	9.00	7.62	7.10
${L_{{{\text{wa}}}}}$ (µH)	0.65	0.65	0.65	0.65
${L_{{{\text{wb}}}}}$ (µH)	0.70	0.70	0.70	0.69
Calculated values				
${R_{{{\text{el}}}}}$ (kΩ)	2.16	6.13	9.95	13.51
${C_{{{\text{el}}}}}$ (pF)	0.24	0.24	0.95	0.95
${C_{{{\text{Pa}}}} = C_{{{\text{Pb}}}}}$ (nF)	80.24	138.14	107.75	83.02
${m_{{{\text{Ca}}}} = m_{{{\text{Cb}}}}}$ (–)	0.84	0.79	0.78	0.82
$R_{{{\text{Pa}}}} = R_{{{\text{Pb}}}}$ (MΩ)	1.01	0.826	0.95	0.47
${m_{{{\text{Ra}}}} = m_{{{\text{Rb}}}}}$ (–)	0.98	0.90	0.88	0.72
$R_{{{\text{Fa}}}} = R_{{{\text{Fb}}}}$ (MΩ)	4.29	1.84	2.46	3.93
${R_{{{\text{elep}}}}}$ (kΩ)	1.09	3.01	5.19	7.25
${C_{{{\text{ep}}}}}$ (pF)	0.49	9.00	7.62	7.10
${R_{{{\text{ep}}}}}$ (Ω)	∞	∞	∞	∞

**Fig. 5:**
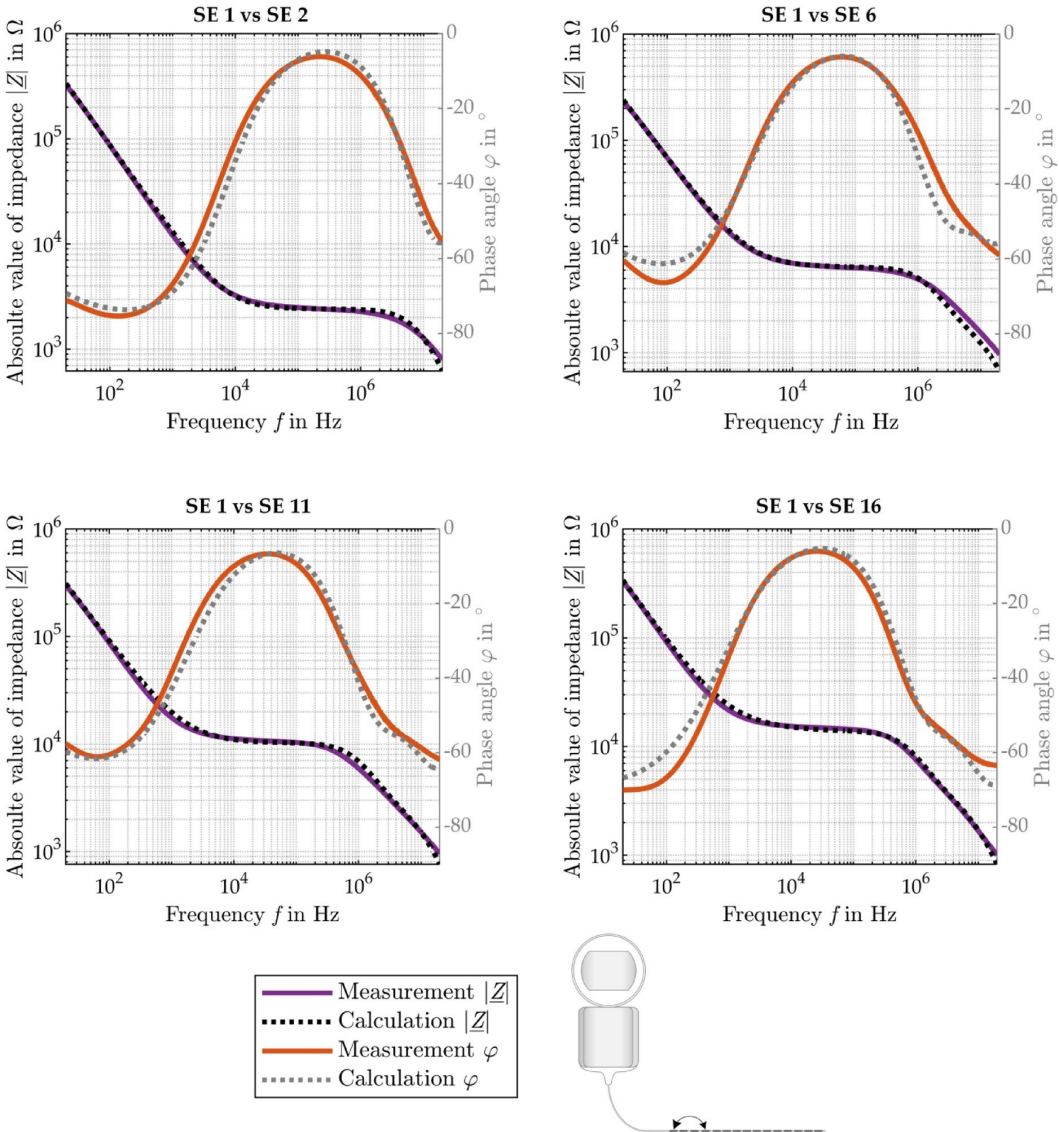
Measured (violet solid line) and modeled (from EEC, black dotted line) absolute value $\left| {\underline {Z} } \right|$ and measured (orange solid line) and calculated (from EEC, gray dotted line) phase angle φ of the complex impedance between two SEs.

Figure 5 exemplary shows the experimental impedance spectroscopic data as well as the absolute value $\left| {\underline {Z} } \right|$ and the phase angle $\varphi$ of the complex impedance modeled with the bipolar EEC from Fig. 1 using the values from Table 1. As can be seen, the EEC model matches the experimental data quite well.

This is quantitatively supported by the MRE across all frequencies of the modeled complex impedance and the absolute value as well as the MAE of the modeled phase angle referenced to the experimental values measured between the most apical SE and all other SEs summarized in Table 2.

**Table 2:** MRE of the modeled complex impedance and modeled absolute value and MAE of the modeled phase angle referenced to the experimental data.

	SE 1 vs. SE 2	SE 1 vs. SE 6	SE 1 vs. SE 11	SE 1 vs. SE 16	Average (SE 1 vs. SE 2 to SE 16)
MRE of absolute value $\overline{e}_{Z}$ (%)	4.19 ± 3.42	5.06 ± 6.65	6.32 ± 5.01	5.02 ± 3.51	5.40 ± 4.46
MAE of phase angle $\overline{e}_{\varphi }$ (°)	2.30 ± 1.61	2.45 ± 2.39	2.24 ± 1.39	2.63 ± 2.19	2.39 ± 1.70
MRE of complex impedance $\overline{e}_{{\underline {Z} }}$ (%)	6.48 ± 3.41	7.68 ± 6.71	8.03 ± 4.79	7.45 ± 4.41	7.56 ± 4.39

### Power analysis in the frequency domin using the bipolar and the monopolar EEC

**Fig. 6:**
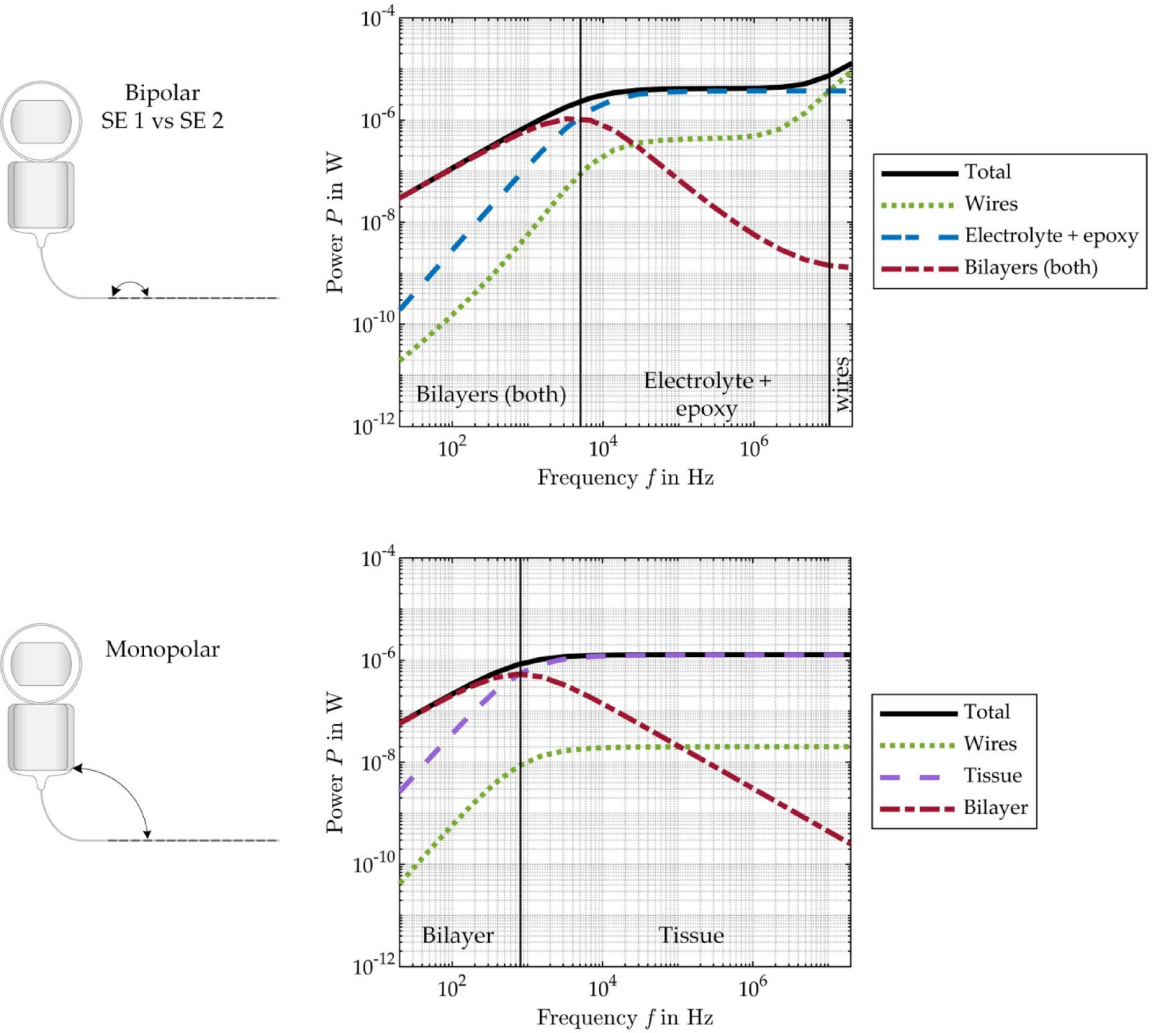
Total power and power distribution over the different CI electrode components and surrounding tissue/material calculated with the EEC models as a function of frequency with a sinusoidal voltage excitation of 100 mV_p_ in bipolar (top) and monopolar (bottom) arrangement.

As illustrated in Fig. 6, the numerically modeled power distribution across the various components of a cochlear implant, in particular the CI electrode and surrounding tissue, is depicted as a function of the frequency of a continuous sinusoidal voltage stimulus, applied to the bipolar (top) and the monopolar (bottom) EEC model. For this purpose, the values from Table 1 (SE 1 vs. SE 2) were used for the elements in both EEC models. Furthermore, a tissue resistance of 7.7 kΩ is assumed in the monopolar EEC model. It is evident from Fig. 6 that the power consumption of the CI electrode initially increases with increasing frequency. Within the low frequency range (bipolar: 20 Hz to 5 kHz, monopolar: 20–800 Hz), the predominant power conversion is located within the bilayers at the interfaces between the SE and the electrolyte. However, with further increasing frequency, the power distribution undergoes a shift, with the majority of the power being dissipated in the surrounding tissue (monopolar) or the electrolyte and epoxy (bipolar). Within the mid frequency range of (bipolar: 5 kHz to 10 MHz, monopolar: 800 Hz to 20 MHz), the total power plateaus and remains relatively constant. While the total power for monopolar stimulation remains constant from 800 Hz onwards and is dominated by the power dissipation in the tissue, the total power for bipolar stimulation increases with increasing frequency from 10 to 20 MHz, with the main part of the power being dissipated in the electrode wires from 10 MHz onwards. At 15 MHz, the power consumption for bipolar stimulation is ten times higher than for monopolar stimulation. The different characteristic frequency ranges for monopolar and bipolar configuration simply result from the different EECs.

### Signal analysis in the time domain

**Fig. 7:**
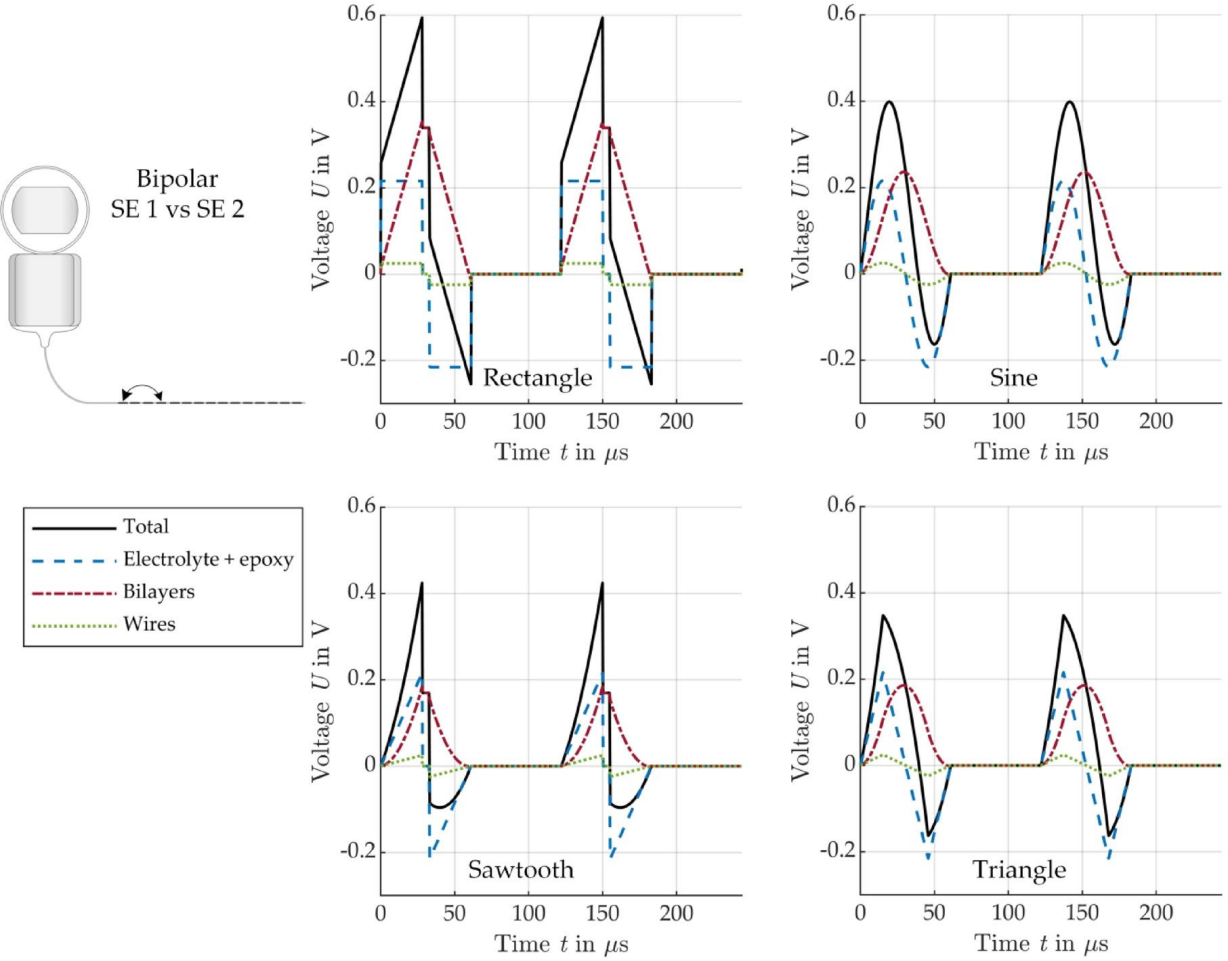
Modeled voltage across the different bipolar EEC components for stimulation with a biphasic current pulse of different shape: rectangle (top left), sine (top right), sawtooth (bottom left), triangle (bottom right), see Fig. 3.

Figure 7 shows the modeled voltage signals across the bipolar EEC components for the four different current stimulation pulse shapes: rectangle, sawtooth, sine and triangle. The voltage across the wires (maximum value of 25 mV), electrolyte and epoxy (maximum value of 216 mV) follows the shape of the current pulse in contrast to the shape of the total voltage that differs from that of the current pulse. The total voltage does not drop to zero between the positive and negative pulse phase, resulting in an asymmetry of the total voltage despite a biphasic current pulse. The asymmetry results from the voltage across the bilayers which remains positive even during the negative current pulse and is almost axisymmetric to the center of the biphasic current pulse.

**Fig. 8:**
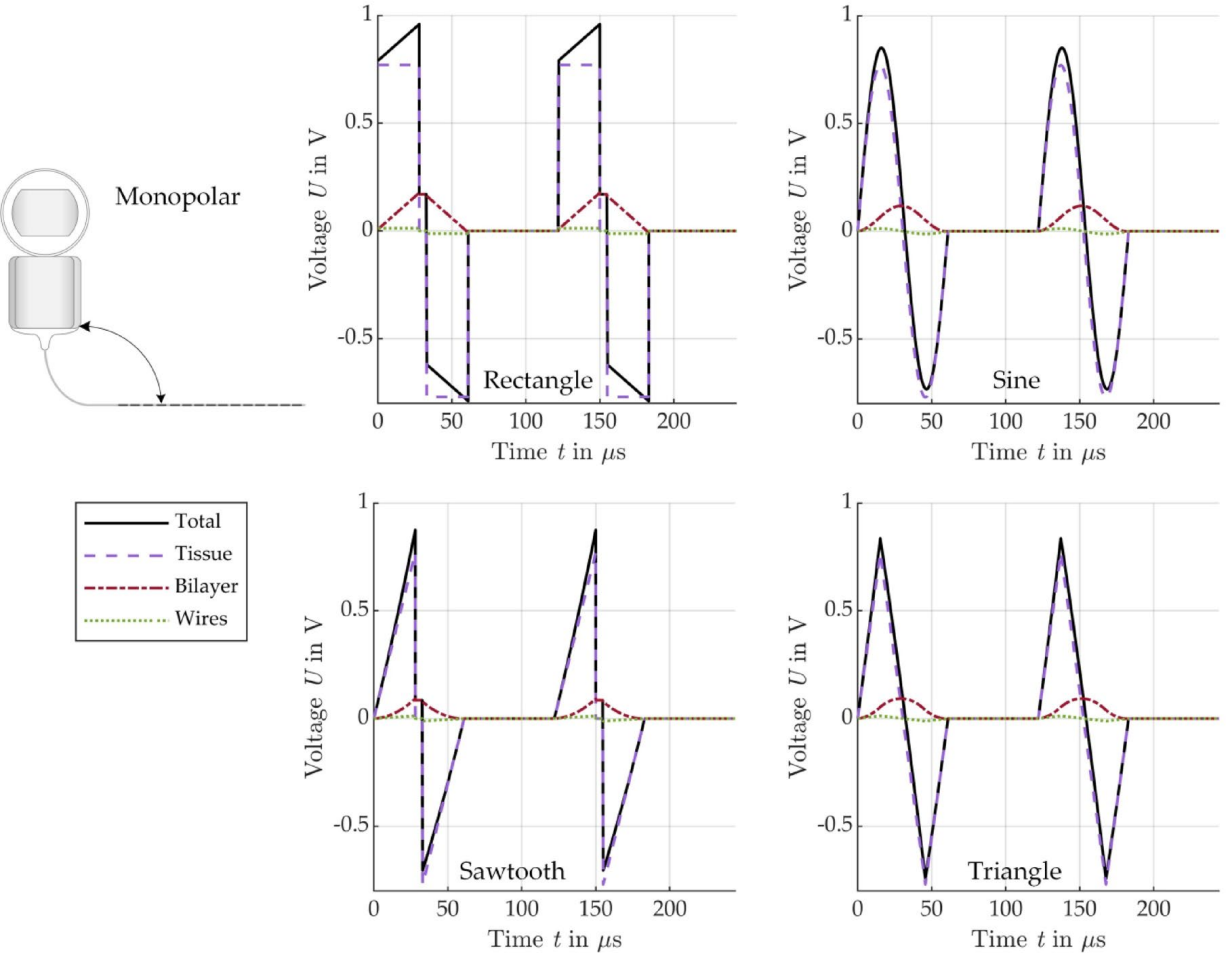
Modeled voltage across the different monopolar EEC components for stimulation with a biphasic current pulse of different shape: rectangle (top left), sine (top right), sawtooth (bottom left), triangle (bottom right), see Fig. 3.

Similar behavior can be seen for monopolar stimulation in Fig. 8. The voltages across wires (maximum value of 12 mV) and tissue (maximum value of 770 mV) again follow the current pulse shape. The voltage across the bilayer corresponds to the behavior of the bilayer during bipolar stimulation. However, the voltage is only half as high compared to the bipolar arrangement since only the bilayer of one SE is considered in the monopolar arrangement, while the possible bilayer at the counter electrode is neglected here. For example, the voltage across the bilayers in the 5 µs pause plateau is 338 mV (bipolar) and 169 mV (monopolar). The asymmetry of the total voltage is therefore less pronounced in the monopolar arrangement.

### Power analysis in the time domain

**Fig. 9:**
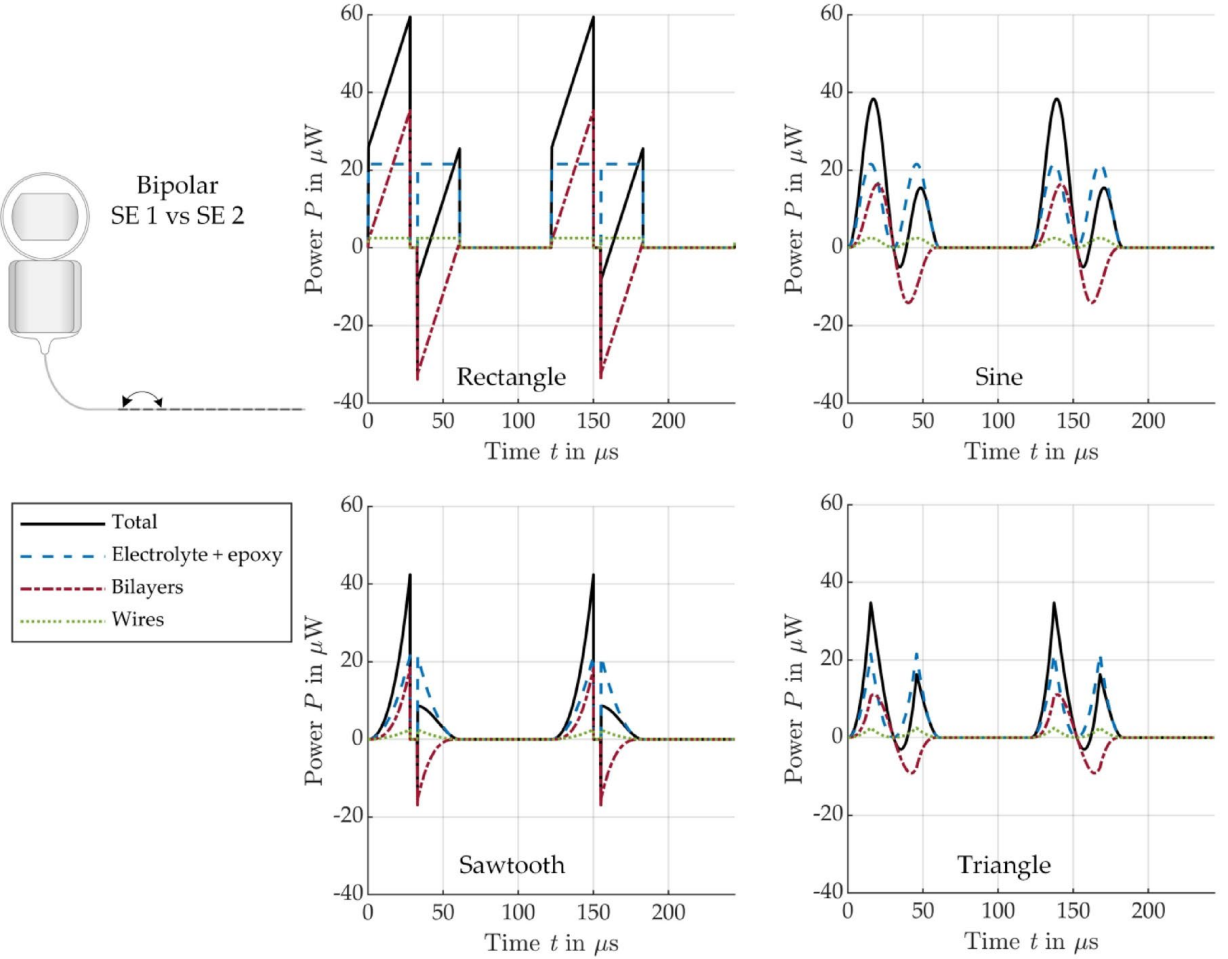
Modeled power conversion in the different bipolar EEC components for stimulation with a biphasic current pulse of different shape: rectangle (top left), sine (top right), sawtooth (bottom left), triangle (bottom right), see Fig. 3.

**Fig. 10:**
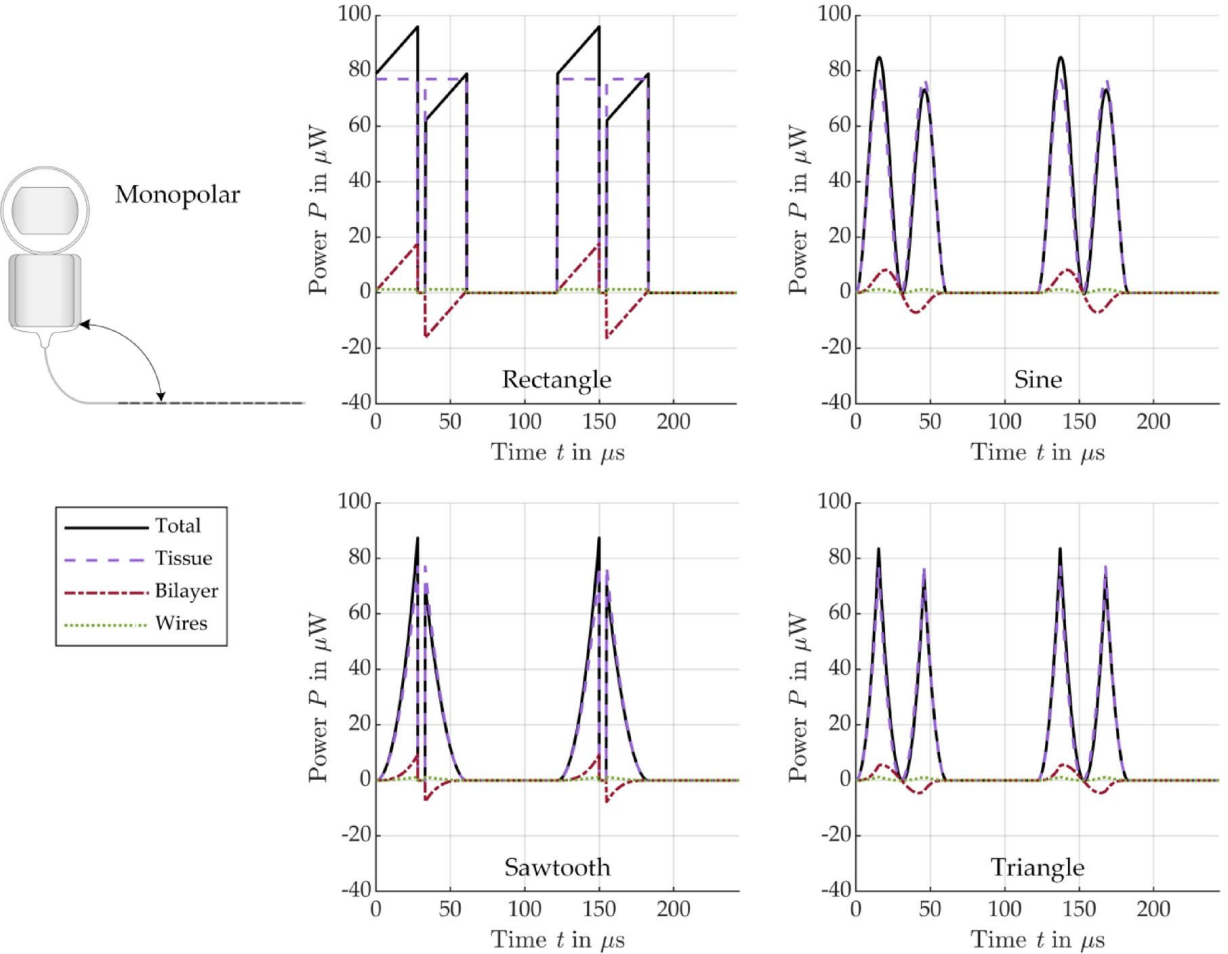
Modeled power conversion in the different monopolar EEC components for stimulation with a biphasic current pulse of different shape: rectangle (top left), sine (top right), sawtooth (bottom left), triangle (bottom right), see Fig. 3.

To assess the energy efficiency of the different signal shapes, Fig. 9 (bipolar) and Fig. 10 (monopolar) show the modeled active power ($P = I \cdot U \cdot cos\left( {\varphi_{u} - \varphi_{i} } \right)$), which is dissipated in the different EEC components.

In all cases considered, it can be seen that the dissipated power in the electrolyte, tissue and wires during the positive pulse phase is exactly the same as during the negative pulse phase. As expected, the shapes of the power curves for these components of the EEC correspond to the squared current pulse shape. The shape of the power dissipated in the bilayers is different. For all pulse shapes, the power dissipation in the bilayers increases slower compared to all the other EEC components and becomes negative during the negative pulse phase. As a result, the total power, is not the same in the two pulse phases and sometimes becomes negative for a short time after the 5 µs pause. The total power only becomes negative in bipolar arrangement, when the negative power in the bilayers has a higher absolute value than the sum of all positive powers in the other EEC components (electrolyte, epoxy and wires) during the negative pulse phase, see Fig. 9.

Table 3 summarizes the energy consumption (integral of power over time) in the individual EEC components during an entire biphasic current pulse, depending on the pulse shape. A detailed distribution of the energies divided into positive and negative pulses can be found in the Supplementary information in Tables S1 to S4.

**Table 3:** Energy consumption of all EEC components during a biphasic stimulation pulse of different shapes.

	Rectangle		Sawtooth		Sine		Triangle	
	nJ	%	nJ	%	nJ	%	nJ	%
Bipolar								
Total	1.43	100	0.48	100	0.78	100	0.52	100
Electrolyte + epoxy	1.21	84.58	0.40	84.58	0.66	84.58	0.44	84.59
Bilayers	0.08	5.69	0.03	5.67	0.04	5.68	0.03	5.68
Wires	0.14	9.74	0.05	9.74	0.08	9.73	0.05	9.73
Monopolar								
Total	4.42	100	1.47	100	2.41	100	1.61	100
Tissue	4.31	97.50	1.44	97.50	2.35	97.50	1.57	97.50
Bilayer	0.04	0.94	0.01	0.94	0.02	0.94	0.02	0.94
Wire	0.07	1.56	0.02	1.56	0.04	1.56	0.03	1.56

## Discussion

In the present study, a new bipolar EEC representing a CI electrode including the surrounding tissue was developed that matches quiet well the experimental data from impedance measurements between two SEs over a wide frequency range. In addition, based on the bipolar EEC, a new monopolar EEC was developed representing a CI electrode including the surrounding tissue when one SE is measured against a counter electrode located outside the cochlea. The two EECs allow for theoretically investigating the power distribution over the different EEC elements and thus over the different components of the CI electrode and the surrounding tissue for different stimulation pulse shapes. Subsequently, the theoretical data allow for assessing the energy efficiency of different stimulation pulse shapes with respect to the energy dissipated in the CI electrode in relation to the tissue.

In detail, the bipolar EEC accurately describes the complex impedance between two SEs with MRE ≤ 8%. In comparison to a previously developed EEC for the complex impedances between neighboring SEs^19^, the new bipolar EEC was extended by the surrounding environment and is therefore able to describe the complex impedance for all SE combinations. This new in silico model was experimentally validated with bipolar impedance spectroscopic measurements using a CI HiFocus SlimJ from Advanced Bionics placed in a saline filled epoxy cylinder used as a straight cochlea phantom. Based on the bipolar EEC, a monopolar EEC was developed to model the stimulation between one SE and a counter electrode located outside the cochlea, including the return pathway through the tissue.

A numerical power analysis in the frequency domain of both EEC models resulted in an optimal frequency range regarding energy efficiency for the stimulation of a CI electrode with a sine voltage (monopolar: above 2 kHz, bipolar: 20 kHz to 10 MHz). In this frequency range, most of the input power is available for stimulating the SGNs and is not dissipated in the wires or the bilayers of the SEs. Above 10 MHz, most of the power is used up in the wires in bipolar arrangement, due to the capacitive coupling that occurs between the wires of the two active SEs, while most of the power still reaches the tissue in monopolar arrangement, see Fig. 6. Since no sine wave is used for excitation in CI, this statement is of course only of academic interest, but already shows how frequency and thus stimulation pulse shape with its characteristic frequencies affect the loss of energy in the CI electrode in relation to the tissue making frequency a crucial parameter for energy efficient stimulation.

Analyzing the voltages across the individual components of the EEC in time domain for different current pulse shapes (rectangle, sawtooth, triangle, sine) showed that the bilayers at the interface between the electrolyte and the SE have a significant influence on total voltage. The bilayers effectively act as an energy storage, due to their polarization capacitance, charging during the positive pulse phase and discharging during the negative pulse phase. This characteristic is also visible when analyzing the power in the time domain, where the power in the bilayers is negative during the negative pulse phase, indicating that energy is being returned to the CI electrode. The four analyzed pulse shapes had a period duration of 61 µs, resulting in a fundamental frequency of 16.4 kHz. According to the frequency analysis, the bilayers should not have a significant effect on the stimulation at this frequency, especially in monopolar arrangement, but the bilayers do not appear to be negligible. Although the bilayers define the shape of the total voltage, the voltages across the tissue and the electrolyte always follow the shape of the stimulating current pulse, which is the reason for using current pulse stimulation. Although the total voltage is asymmetrical, the voltage across the tissue is still symmetrical as intended.

According to the energy analysis in time domain, most of the energy introduced during stimulation actually reaches the tissue surrounding the CI electrode (monopolar: 97.5%, bipolar: 84.5%). The relative energy distribution across the individual components of the EECs and thus across the individual parts of the CI electrode is the same for all pulse shapes. However, the absolute energy consumption for monopolar stimulation is significantly higher than for bipolar stimulation when stimulating with the same current amplitude, as the tissue resistance is significantly higher in monopolar than in bipolar arrangement due to the large distance from the SE to the counter electrode located outside the cochlea. Conversely, monopolar stimulation would require less current, and therefore resulting in lower energy consumption, than bipolar stimulation to achieve the same voltage across the tissue. Several studies have shown that stimulation of the SGNs is more efficient in monopolar configuration requiring lower current to reach stimulation thresholds than in bipolar configuration^32,33^. Nevertheless, it has to be mentioned that our model assumes a very high tissue resistance of 7.7 kΩ, based on literature values^30^, and that the tissue resistance might be significantly lower in reality, which needs to be verified in future studies. As expected, a comparison of the absolute values of the total energies shows that for the same current amplitude, the signals with lower slope require less energy than the typically used rectangular signal. The sawtooth requires only 33%, the triangle 36% and the sine 55% of the energy that a rectangular pulse requires in both stimulation configurations, monopolar and bipolar. It is interesting to note, however, that although the integral of a phase of the sawtooth current pulse is half as large as that of the rectangular pulse, the energy requirement is only one third, which is due to the fact that the capacitive bilayers cannot follow the current when increasing fast.

It is important to note, that the results of this study exclusively relate to the behavior of the CI electrode and its interaction with the surrounding tissue. In order to obtain a comprehensive picture of the energy efficiency of a CI, all electrically relevant components of the CI, including the pulse generator (current source), would have to be included in the analysis. For example, Vanpoucke et al. have integrated the current source of the CI in their EEC with a decoupling capacitance between the current source and SE, also used by Aebischer et al.^14,17^. Similar to the Vanpoucke model, the electrical pathway between the SE and the counter electrode in the presented monopolar EEC was simplified as a tissue resistance neglecting a possible formation of a bilayer at the counter electrode. In the present study, this tissue resistance was estimated on the basis of studies by Hu et al. and Wimmer et al., who have both measured the impedance in monopolar configuration in CI patients at different times after implantation^30,31^. While Vanpoucke et al. and other studies model the bilayer with linear, frequency-independent electrical elements, this study used a non-linear, frequency-dependent polarization capacitance and a non-linear, frequency-dependent polarization resistance to model the bilayer between the SEs and the electrolyte more accurately^14–17^. However, the non-linearity of the bilayer was only validated for small voltages yet. Further non-linearities arising from higher voltages were not considered. Another important aspect of energy efficient stimulation of SGNs that is not discussed here, as it would go beyond the scope of this work, is, how more refined signal shapes of a stimulation pulse affects stimulation of SGNs. However, the method presented here enables a more sophisticated framework for analysis of pulse shapes in future studies.

Although the extended bipolar EEC was only experimentally validated for one type of CI from one manufacturer, it can be assumed that it is also applicable for other CIs, as the underlying EEC for neighboring SEs has already been experimentally validated in previous work^19^ with four CIs from four leading CI manufacturers MED-EL Medical Electronics GmbH (MED-EL, Innsbruck, Austria), Advanced Bionics LLC (AB, Valencia, CA, USA), Oticon Medical/Neurelec SAS (Oticon, Vallauris, France) and Cochlear Ltd. (Cochlear, Sydney, Australia) varying in design and distance between SEs. In addition, all CIs have a similar design and principally can be divided into the same components—the wires, the SEs with bilayers and the surrounding tissue as already shown. One important limitation of the present work is that the extended bipolar EEC was just validated in a straight epoxy cochlea phantom. For final validation of both EEC models, further tests with other CI types from different manufacturers as well as measurements in human cochleae appear necessary, but were out of scope in this study.

Nevertheless, the presented EEC models allow for an easy in silico characterization of different stimulation pulse shapes regarding power distribution over the different components of the CI electrode and surrounding tissue and thus the energy loss in the CI electrode in relation to the tissue. Furthermore, in future combination with a model of the nerve fiber^8,34^, the two EEC models could help to optimize the stimulation of SGNs.

## Conclusion

In the present study, two EEC models were developed and experimentally validated with high accuracy that describe the frequency-dependent impedance of a cochlear implant for bipolar and monopolar stimulation with different biphasic current pulse shapes. The main results can be summarized as follows:

1. The bipolar EEC describes the complex impedance between two different stimulation electrodes of a cochlear implant with high accuracy of ≥ 92% over a wide frequency range.
2. There is an optimal frequency range with minimum energy loss in the CI electrode for an efficient transfer of signal energy into the tissue (bipolar: 20 kHz to 20 MHz, monopolar: above 2 kHz).
3. The bilayers have a significant influence on the total voltage signal across the CI electrode, but the voltages across the tissue and the electrolyte always follow the shape of the stimulating current pulse. Also, the bilayers were identified as energy storages charging during the positive pulse phase, which release energy during the negative pulse phase.
4. For all investigated pulse shapes, the majority of energy introduced during stimulation actually reaches the tissue surrounding the CI electrode (monopolar: 97.5%, bipolar: 84.5% of the total energy) and only a small fraction of the total energy is lost by wires and bilayers.
5. The sawtooth requires only 33%, the triangle 36% and the sine 55% of the energy that a rectangular pulse with the same current amplitude requires in monopolar and bipolar stimulation configurations.

## Electronic supplementary material

Below is the link to the electronic supplementary material.

Supplementary Material 1

## Data Availability

The datasets used and/or analyzed during the current study are available from the corresponding author upon reasonable request.
