# Supplementary material for: Electrical equivalent circuit for analyzing the effect of signal shape on power distribution in cochlear implant electrodes and surrounding tissue
Source: Sci Rep. 2025 Jun 20;15:20136. doi: 10.1038/s41598-025-04840-5 (PMC12181362; doi:10.1038/s41598-025-04840-5)
Supplement: Supplementary file 1 — Supplementary Material 1 [file 41598_2025_4840_MOESM1_ESM.docx]

Supplementary information


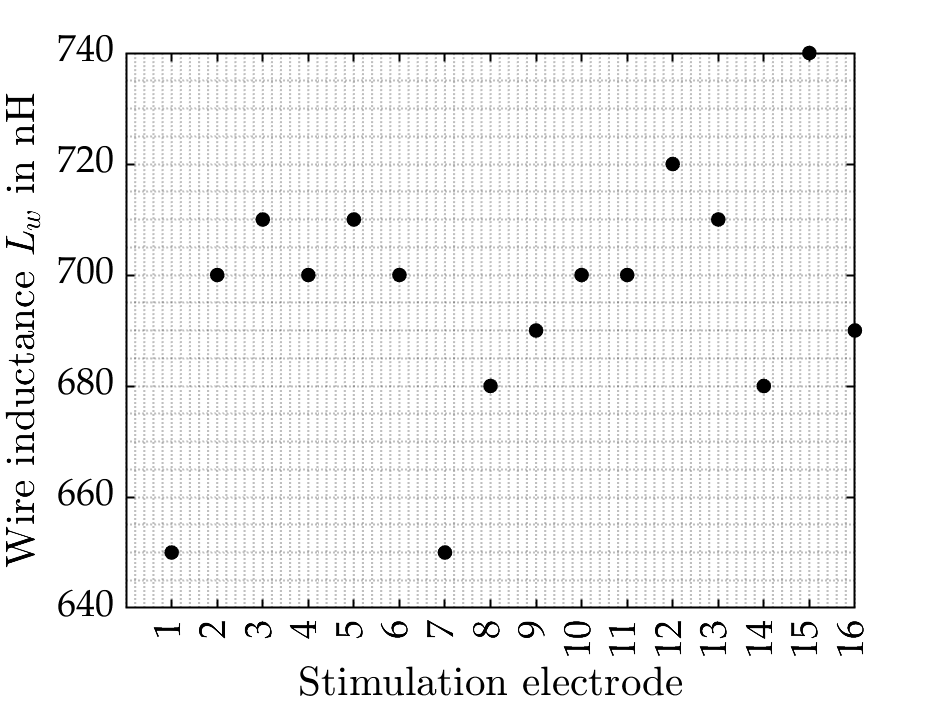


Figure S1: Measured inductances for each stimulation electrode of a CI HiFocus SlimJ measured with an LCR meter

**Table S1.** Distribution of the energy during a rectangular current stimulation pulse

|  |  | **Positive pulse duration** | | **Negative pulse duration** | | **Biphasic pulse duration** | |
| --- | --- | --- | --- | --- | --- | --- | --- |
|  |  | **nJ** | **%** | **nJ** | **%** | **nJ** | **%** |
| **Monopolar** | **Total** | 2.45 | 100.00 | 1.97 | 100.00 | 4.42 | 100.00 |
|  | **Tissue** | 2.16 | 88.05 | 2.16 | 109.24 | 4.31 | 97.50 |
|  | **Bilayer** | 0.26 | 10.55 | -0.22 | -10.98 | 0.04 | 0.94 |
|  | **Wires** | 0.03 | 1.41 | 0.03 | 1.75 | 0.07 | 1.56 |
| **Bipolar** | **Total** | 1.19 | 100.00 | 0.24 | 100.00 | 1.43 | 100.00 |
|  | **Electrolyte + epoxy** | 0.60 | 50.80 | 0.60 | 252.36 | 1.21 | 84.58 |
|  | **Bilayers** | 0.52 | 43.35 | -0.43 | -181.42 | 0.08 | 5.69 |
|  | **Wire** | 0.07 | 5.85 | 0.07 | 29.06 | 0.14 | 9.74 |

**Table S2.** Distribution of the energy during a sawtooth current stimulation pulse

|  |  | **Positive pulse duration** | | **Negative pulse duration** | | **Biphasic pulse duration** | |
| --- | --- | --- | --- | --- | --- | --- | --- |
|  |  | **nJ** | **%** | **nJ** | **%** | **nJ** | **%** |
| **Monopolar** | **Total** | 0.80 | 100.00 | 0.68 | 100.00 | 1.47 | 100.00 |
|  | **Tissue** | 0.72 | 90.23 | 0.72 | 106.05 | 1.44 | 97.50 |
|  | **Bilayer** | 0.07 | 8.32 | -0.05 | -7.74 | 0.01 | 0.94 |
|  | **Wire** | 0.01 | 1.44 | 0.01 | 1.70 | 0.02 | 1.56 |
| **Bipolar** | **Total** | 0.36 | 100.00 | 0.12 | 100.00 | 0.48 | 100.00 |
|  | **Electrolyte + epoxy** | 0.20 | 56.43 | 0.20 | 168.80 | 0.40 | 84.58 |
|  | **Bilayers** | 0.13 | 37.07 | -0.11 | -88.24 | 0.03 | 5.67 |
|  | **Wires** | 0.02 | 6.50 | 0.02 | 19.44 | 0.05 | 9.74 |

**Table S3.** Distribution of the energy during a triangle current stimulation pulse

|  |  | **Positive pulse duration** | | **Negative pulse duration** | | **Biphasic pulse duration** | |
| --- | --- | --- | --- | --- | --- | --- | --- |
|  |  | **nJ** | **%** | **nJ** | **%** | **nJ** | **%** |
| **Monopolar** | **Total** | 0.87 | 100 | 0.73 | 100 | 1.61 | 100 |
|  | **Tissue** | 0.78 | 89.64 | 0.78 | 106.88 | 1.57 | 97.50 |
|  | **Bilayer** | 0.08 | 8.93 | -0.06 | -8.59 | 0.02 | 0.94 |
|  | **Wire** | 0.01 | 1.43 | 0.01 | 1.71 | 0.03 | 1.56 |
| **Bipolar** | **Total** | 0.40 | 100 | 0.12 | 100 | 0.52 | 100 |
|  | **Electrolyte + epoxy** | 0.22 | 54.83 | 0.22 | 184.99 | 0.44 | 84.59 |
|  | **Bilayers** | 0.16 | 38.86 | -0.13 | -106.28 | 0.03 | 5.68 |
|  | **Wires** | 0.03 | 6.31 | 0.03 | 21.29 | 0.05 | 9.73 |

**Table S4.** Distribution of the energy during a sine current stimulation pulse

|  |  | **Positive pulse duration** | | **Negative pulse duration** | | **Biphasic pulse duration** | |
| --- | --- | --- | --- | --- | --- | --- | --- |
|  |  | **nJ** | **%** | **nJ** | **%** | **nJ** | **%** |
| **Monopolar** | **Total** | 1.32 | 100 | 1.09 | 100 | 2.41 | 100 |
|  | **Tissue** | 1.17 | 89.06 | 1.17 | 107.72 | 2.35 | 97.50 |
|  | **Bilayer** | 0.13 | 9.52 | -0.10 | -9.44 | 0.02 | 0.94 |
|  | **Wire** | 0.02 | 1.42 | 0.02 | 1.72 | 0.04 | 1.56 |
| **Bipolar** | **Total** | 0.62 | 100 | 0.16 | 100 | 0.78 | 100 |
|  | **Electrolyte + epoxy** | 0.33 | 53.31 | 0.33 | 204.60 | 0.66 | 84.58 |
|  | **Bilayers** | 0.25 | 40.55 | -0.21 | -128.14 | 0.04 | 5.68 |
|  | **Wires** | 0.04 | 6.13 | 0.04 | 23.54 | 0.08 | 9.73 |
